# Engineering Tissue Fabrication With Machine Intelligence: Generating a Blueprint for Regeneration

**DOI:** 10.3389/fbioe.2019.00443

**Published:** 2020-01-10

**Authors:** Joohyun Kim, Jane A. McKee, Jake J. Fontenot, Jangwook P. Jung

**Affiliations:** ^1^Center for Computation and Technology, Louisiana State University, Baton Rouge, LA, United States; ^2^Department of Biological Engineering, Louisiana State University, Baton Rouge, LA, United States

**Keywords:** machine learning, bioprinting, tissue engineering, cardiovascular, machine intelligence

## Abstract

Regenerating lost or damaged tissue is the primary goal of Tissue Engineering. 3D bioprinting technologies have been widely applied in many research areas of tissue regeneration and disease modeling with unprecedented spatial resolution and tissue-like complexity. However, the extraction of tissue architecture and the generation of high-resolution blueprints are challenging tasks for tissue regeneration. Traditionally, such spatial information is obtained from a collection of microscopic images and then combined together to visualize regions of interest. To fabricate such engineered tissues, rendered microscopic images are transformed to code to inform a 3D bioprinting process. If this process is augmented with data-driven approaches and streamlined with machine intelligence, identification of an optimal blueprint can become an achievable task for functional tissue regeneration. In this review, our perspective is guided by an emerging paradigm to generate a blueprint for regeneration with machine intelligence. First, we reviewed recent articles with respect to our perspective for machine intelligence-driven information retrieval and fabrication. After briefly introducing recent trends in information retrieval methods from publicly available data, our discussion is focused on recent works that use machine intelligence to discover tissue architectures from imaging and spectral data. Then, our focus is on utilizing optimization approaches to increase print fidelity and enhance biomimicry with machine learning (ML) strategies to acquire a blueprint ready for 3D bioprinting.

## Introduction

Tissue Engineering (TE) has advanced over the last few decades to tackle challenging problems in tissue regeneration (Shafiee and Atala, 2017; Armstrong and Stevens, 2019). Of many available tools and methods for tissue and organ fabrication, 3D bioprinting (3DBP) has been widely applied to create tissue-specific microenvironments and patient-specific organs (Giannitelli et al., 2015; Jung et al., 2016; Morss Clyne et al., 2019; Tamay et al., 2019). Recent examples of 3D bio-printed tissues include a multicellular human scale 3DBP platform (Kang et al., 2016), thick engineered vessels (Kolesky et al., 2016), convoluted renal proximal tubules (Homan et al., 2016), a vascularized alveolar model (Grigoryan et al., 2019), a bioprosthetic ovary model (Laronda et al., 2017), a neonatal scale human heart with vasculature and heart valve (Lee et al., 2019), a personalized perfusable cardiac patch (Noor et al., 2019), and printing stem cell-derived organoids as a building block (Skylar-Scott et al., 2019). Despite the advancement evidenced by literature, significant challenges are still ahead to effectively handle the complexity that originates from native tissue components and architecture (Ogle et al., 2016; Ghaemi et al., 2019). While the majority of 3DBP publications focused on printing methods (Cui et al., 2017) and bioinks (Valot et al., 2019), only a few focused on generating a blueprint for regeneration (Jung et al., 2012; Hanson et al., 2013; Gao et al., 2017).

**Figure 1:**
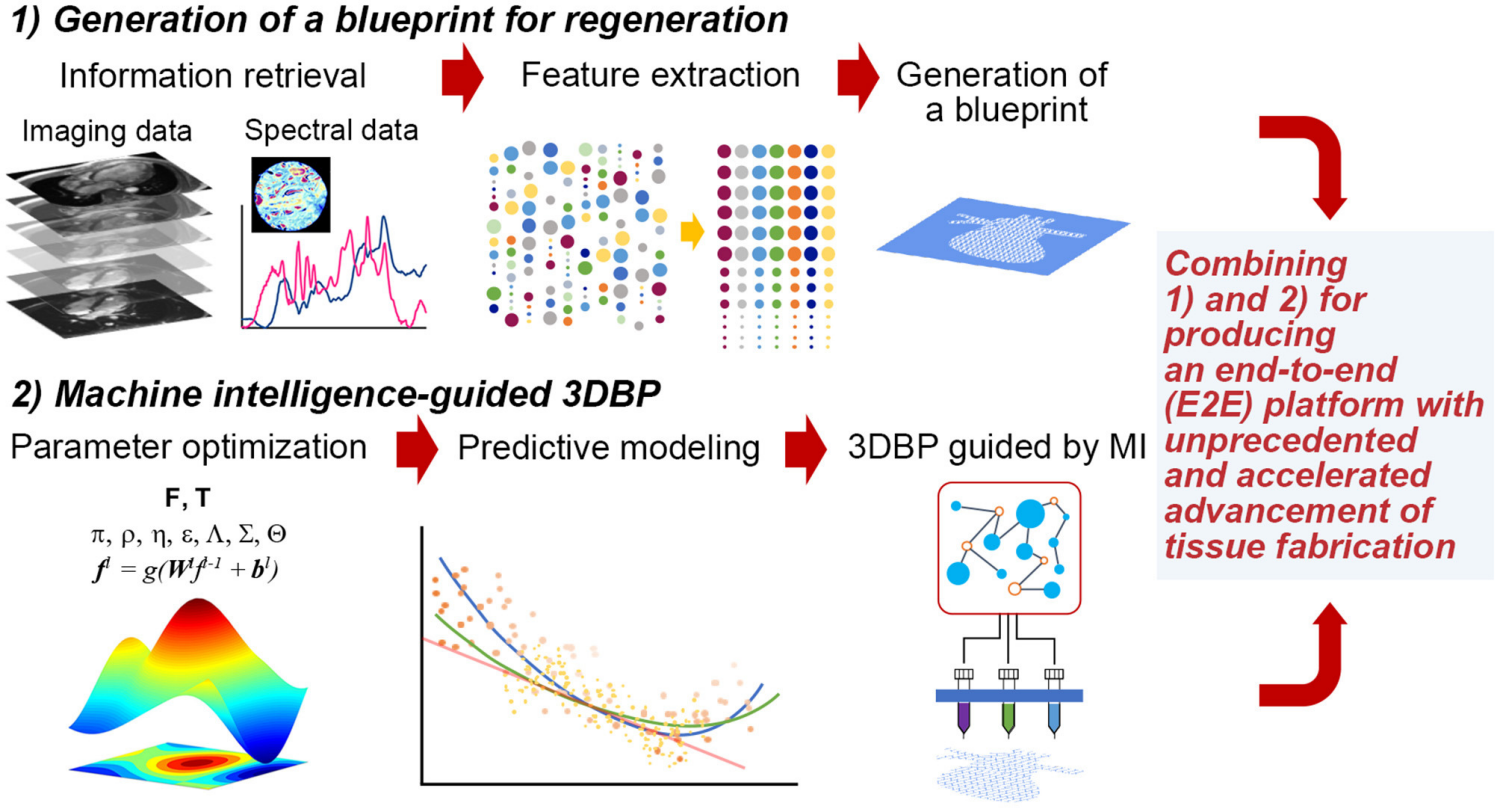
Hypothetical stages to fabricate functional tissues with 3DBP guided by MI. (1) Generation of a blueprint for regeneration: We may acquire data from publicly available database and experimental data from imaging (e.g., microCT or MRI) and spectroscopy (e.g., FT-IR, adapted with permission from Berisha et al., 2019). Then, acquired data will be processed to extract specific features that we can translate into a blueprint. (2) MI-guided 3DBP: To fabricate an organ-size, functional tissue from a complex blueprint, we need to optimize printing parameters leveraging MI. This approach benefits from the capacity of identifying complex data patterns to predict certain parameter space, which may not be possible to obtain without MI and which may accelerate the production of functional tissues.

Thus, it is imperative to collect ample data to extract features that can be successfully translated to a blueprint for 3DBP. In addition, it is desirable to systematically optimize printing parameters and bioink properties to generate such a blueprint. For such a case, Design of Experiment (DOE) approaches are common statistical quality control techniques, utilizing systematic randomization to inform experiment planning, execution, as well as model fitting of the results. Although DOE approaches are widely utilized for optimization (Allen, 2010), these statistical methods may not be suitable to process high-dimensional imaging data and prediction of such high-dimensional data with analytical models. Rather, it would be appropriate to exploit machine intelligence (MI) to perform such inherently complex tasks. The following sections discuss (1) the extraction of information from publicly accessible texts, images and spectral data and (2) optimization with statistical methods, computer algorithms and MI (Figure 1). The future of TE requires more robust guiding principles and templates for regeneration (Williams, 2019) and a reproducible workflow that is not contingent on human expertise (Armstrong and Stevens, 2019).

Generating a blueprint for 3DBP via a systematic strategy will be particularly useful in several aspects (Murphy et al., 2019). Many current 3DBP applications aim to fabricate exact replicates of target tissue, which is difficult due to technological and monetary limits; hence, the degree to which increased complexity leads to increased functionality is unknown and should be investigated. Systematic automation via MI for blueprint design could be useful in 3DBP to better inform personalized medicine via understanding how to determine variable values that ensure successful clinical outcomes. This automation can reduce the cost of tissue design and fabrication logistics for patient-personalized tissues. In addition, processes involved in 3DBP can be standardized to minimize the expense of resources required for blueprint and construct development, which in turn, could streamline clinical processes in order to faster produce 3DBP tissues for patients who need them.

## Automatic Extraction of Existing Knowledge and Pattern Discovery for Generating A Blueprint

### Information Retrieval (IR)

Most of the existing methods of IR have been developed by focusing on a specific modality, rather than a wholistic multi-modal method. Examples include analysis of image data with computer vision techniques (Litjens et al., 2017; Brent and Boucheron, 2018; Carin and Pencina, 2018). IR in biomedical applications will be beneficial for perception problems (i.e., observing images) along with reasoning, under a common platform like human intelligence (Hassabis et al., 2017). Recently, robust and powerful deep learning (DL) models have emerged, offering novel neural models for complex problems, including machine reasoning tasks such as entailment and abstractive summarization (Miller et al., 2016; Devlin et al., 2018), which deal with multi-modality of images altogether. What we expect is various emerging integrative strategies to combine information at different length scales and dimensionality to produce tissue constructs suitable for 3DBP (Del Sol et al., 2017).

### Imaging Data Retrieval and Analysis

One can easily detect characteristics of tissue under investigation and additionally discover hidden features with image analysis, often via machine learning (ML). Although collecting existing image data is the first step, currently existing IR systems are less effective for customized functionalities for tissue image data. Instead, online image data are collected manually or using relatively simple text-based engines. Examples are Yale Image Finder (Xu et al., 2008), Cell Image Library (Orloff et al., 2013), Open Microscopy Environment (Goldberg et al., 2005), and Allen Cell Explorer (Johnson et al., 2017) to name a few, which are resources to be utilized by TE researchers.

Without constructing such a database or retrieval service, one can directly extract information from image data taken with native tissue samples. Microscopy is one of the most popular methods for TE (Dhulekar et al., 2016; Buggenthin et al., 2017; Liang et al., 2017; Brent and Boucheron, 2018; Christiansen et al., 2018; Nitta et al., 2018; Rivenson et al., 2019; Vu et al., 2019), and visual images are also demonstrated to be useful (Gholami et al., 2018). Non-linear imaging methods are also actively developed, including multiphoton (Kistenev et al., 2019) and second harmonic generation (SHG) microscopy, allowing for the visualization of tissue structure and permitting imaging of samples without labeling (Hanson et al., 2013). X-ray microCT, utilized for additive manufacturing (Du Plessis et al., 2018), was demonstrated to be a viable technique for 3D histology (Katsamenis et al., 2019). Magnetic resonance imaging (MRI) is one of the most popular methods used for TE (Jackson et al., 2017). FTIR and Raman microspectroscopy are underexplored techniques in TE, presumably due to their low-resolution nature, but the resulting molecular vibrational or rotational modes can be used as a biochemical fingerprint to characterize tissues (Chen et al., 2012; LeCun et al., 2015; Schmidhuber, 2015; Albro et al., 2018; Gupta et al., 2019; Li et al., 2019; Marzi et al., 2019). Mass spectrometry is used for spatial localization of collagen and elastin in tissue samples (Angel et al., 2018).

Acquisition of imaging and spectral data needs additional steps to instruct 3DBP processes. This implies a significant gap in between data retrieval and generation of tissue blueprints. For examples, imaging structural information of tissue (i.e., extracellular matrix proteins), detailed biochemical information (i.e., via fluorescence microscopy and SHG), tissue anatomy and overall architecture (i.e., using microCT and MRI) need to be combined systematically to generate a collective template for 3DBP. Multi-modality imaging is a way to obtain tissue information, but often requires specific computer programs as well as dedicated imaging techniques (Meng et al., 2017; Guo et al., 2018; Stamatelos et al., 2019). If the abovementioned steps can be streamlined to extract only necessary information for a specific modality of 3DBP (a type of 3D bio-printer), we can reduce efforts and resources. Technological and economical limitations may require a departure from biomimicry in the process of enhancing tissue function, tissue scale or printing throughput (Murphy et al., 2019). It is possible that an MI/ML-based training or model can reduce the gap significantly. The outcome of such MI/ML-based model will include information on biomaterials, their coordinates and overall shape with optimized structural properties. In addition, further information needs to be augmented to reduce the gap between information of tissue microenvironments and the tissue properties at the scale that the 3DBP modality can fabricate. For example, cell adhesion via integrin cannot be specifically fabricated via an extrusion-based 3DBP, but we can to add ECM proteins to mimic cell-matrix interactions. After such a model is established, MI/ML can solve these problems faster without introducing any bias or human errors and further optimize parameters from blueprint to final 3DBP product.

### Pattern Discovery and Translation to a Blueprint for 3DBP

The next step is to unravel hidden or complex patterns from collected information. DL, after being rebranded with breakthroughs for training of deep neural network models, has been incredibly successful for a wide range of pattern discovery uses, which is the main realm of ML (LeCun et al., 2015; Schmidhuber, 2015). Recently, a great number of ML tools have been developed, allowing efficient data mining and predictive models. DL outperforms other methods for image-related problems (Litjens et al., 2017) because of its inherent architecture and learning mechanisms, which are effective for complex, high-dimensional data and computational scalability in analysis.

Pattern discovery can be grouped into (i) ML methods directly targeting imaging data (Brent and Boucheron, 2018; Casiraghi et al., 2018; Gupta et al., 2019; Kistenev et al., 2019; Li et al., 2019; Rivenson et al., 2019; Vu et al., 2019), (ii) ML-based predictive modeling for TE scaffolds (Buggenthin et al., 2017; Tanaka et al., 2017; Chaudhury et al., 2018; Nitta et al., 2018; Marzi et al., 2019; Waisman et al., 2019), and (iii) a broad range of bioinformatics such as network analysis (Camacho et al., 2018). Specifically, several studies are (i) predicting tissue properties with DL from images or experimental observations (Liang et al., 2017; Brent and Boucheron, 2018; Kusumoto et al., 2018; Berisha et al., 2019; Gupta et al., 2019; Kistenev et al., 2019; Lutnick et al., 2019; Rivenson et al., 2019; Vu et al., 2019; Xie et al., 2019), (ii) classifying tissue type, state, and material properties with various ML methods (Casiraghi et al., 2018; Hailstone et al., 2018; Li et al., 2019), (iii) integrating multiple imaging platforms and experiments (Heredia-Juesas et al., 2018), (iv) modeling tissues for pattern discovery and predictive modeling (Bilgin et al., 2010; Yener, 2016; Kusumoto et al., 2018), and (v) extracting information from images for TE (Gholami et al., 2018).

Finally, MI algorithms, including methods leveraging ML, were recently applied to optimizing parameters for 3D printing (Gardner et al., 2019; Menon et al., 2019). Considering the complexity of these additive manufacturing techniques and their potential application to tissue fabrication, it is not surprising to find various methods ranging from biologically inspired ones such as genetic algorithms (GA, which mimic the process of natural selection, de Castro, 2007; Paszkowicz, 2009) to statistical and probabilistic algorithms. They could be grouped as (i) optimal design methods with DOE and its variants such as Taguchi method (Mohamed et al., 2016; Scaffaro et al., 2017; Yousefi et al., 2019), (ii) optimization with population-based methods (Rahmani-Monfared et al., 2013; Asadi-Eydivand et al., 2016; Rao and Rai, 2016; Heljak et al., 2017; Abdollahi et al., 2018), and (iii) problem specific approaches often facilitated by ML (Cheheltani et al., 2012; Farzadi et al., 2015; Tiwari et al., 2015; Langelaar, 2016; Querido et al., 2017; Saadlaoui et al., 2017; Gholami et al., 2018; Shi et al., 2018, 2019; Menon et al., 2019; Zhang et al., 2019; Zohdi, 2019). Despite the abovementioned progress, there is still a significant gap in combining data-to-blueprint translation (Figure 1), which is the process of extracting information from data and incorporating the information in a blueprint file [e.g., CAD (computer-aided design) files] for 3DBP.

## Optimization of Complex 3DBP

### Challenges in Optimizing 3DBP Parameters

In order to produce a 3DBP construct for translational and clinical applications, we need to consider how the generated blueprint from IR is fabricated without compromising fidelity and functionality in TE applications. Assessing the print fidelity and biomimicry of a construct for every possible print parameter combination would be impractical to explore due to time and availability of resources. Systematic optimization methods can aid in the investigation of vast and complicated parameter spaces, and we found a small number of cases that apply systematic optimization to 3DBP procedures (summarized in Table 1 and Tables S1–S3).

**Table 1:** Summary^#^ of 3DBP optimization in improving print fidelity, biomimicry, and integrated approaches.

**Optimization objective**	**Type of printing**	**Optimization method**	**References**
Print fidelity	Piezoelectric drop-on-demand (DOD) bioprinting	Multi-objective optimization (MOO) method	Shi et al., 2019
	Extrusion-based bioprinting	Expert-guided optimization (EGO) method	Abdollahi et al., 2018
	Extrusion-based bioprinting	Hierarchical machine learning (HML)	Menon et al., 2019
	Fused filament fabrication 3D printing	Machine learning technique	Gardner et al., 2019
Biomimicry	Extrusion-based bioprinting	^$^Bioink formulations	Dubbin et al., 2017; Abdollahi et al., 2018; Darnell et al., 2018; Peak et al., 2019; Takebe and Wells, 2019
	Extrusion-based bioprinting	^$^Porosity and flow conditions	Trachtenberg et al., 2018
	3D Bioplotting	^$^Porosity, pore size and interconnectivity	Diaz-Gomez et al., 2019
	Fused deposition modeling (FDM)	Finite element analysis (FEA) with genetic algorithm (GA)	Heljak et al., 2017
Integrated	Extrusion-based bioprinting	Parameter optimization index (POI)	Webb and Doyle, 2017; Giuseppe et al., 2018
	3D Bioplotting	I-optimal, split-plot DOE, and COMSOL-based FEA	Uth et al., 2017
	Additive manufacturing and thermally induced phase separation (TIPS)	I-optimal DOE and the response surface analysis	Yousefi et al., 2019

# *Further details with print parameters and response variables are provided in Tables S1–S3*.

$ *Features not strictly pertaining to optimization methods*.

### Efforts to Increase Print Fidelity in 3DBP

The optimization of print fidelity refers to finding the ideal combination of printing parameters and their corresponding values that promote accurate reflections of blueprints. Some systematic approaches have been attempted without incorporating the utility of MI. One method, termed the multi-objective optimization (MOO) method, used a hybrid multiple subgradient descent bundle (MSGDB) method in conjunction with an Adam algorithm to reduce satellite droplet formation and increase printing precision and stability in piezoelectric drop-on-demand bioprinting (Shi et al., 2019). MSGDB is a gradient-based method for which a candidate descent direction is chosen by combining descent directions calculated with each objective in the case of MOO. The Adam algorithm (Kingma and Ba, 2014) is a recently introduced gradient-based method for stochastic optimization, which is based on adaptive estimation of the first and the second moment of the gradient and widely popular in applications including DL with better performance, stability, and comparable computational cost. The Adam algorithm computes individual adaptive learning rates for parameters and estimates the first and second moment of gradients to optimize weights matrices. Parameters for satellite droplet formation (applied voltage, viscosity of bioink, surface tension, nozzle radius) were processed through a fully connected neural network to reduce the occurrence of satellite droplets and optimize precision, stability, and droplet formation.

A method termed Expert-Guided Optimization (EGO) for 3DBP employs a hill-climbing algorithm to optimize print fidelity through the exploration of print parameters (Abdollahi et al., 2018). Response variables, such as stringiness, infill, and layer fusion, are graded to determine the print accuracy of the 3DBP prints. The EGO method requires the experimenter to identify the parameter space and select the parameters and factor levels that will be considered during the optimization process. Next, random combinations of the selected parameters are tested and evaluated. The best run is then identified, and new combinations of parameters are generated that are similar to the best run but with slightly different parameter values. Then the process is repeated with the new best run, until a combination with optimal stringiness, infill, and layer fusion is identified (termed hill-climbing). After each hill-climbing iteration, the experimenter can decide whether to adjust the considered parameters and/or whether to try different parameter factor levels. While this optimization method is structured and efficient, it poses a risk of local rather than global parameter optimization. Building off of the foundation created in the EGO study, another investigation employed a hierarchical ML (HML) approach with freeform reversible embedding (FRE) to 3D print silicone elastomer into a hollow cylinder tube (Menon et al., 2019). Similar to the EGO method, the HML method aimed to find the combination of parameters that would produce a 3DBP construct with optimal stringiness, infill, and layer fusion. Surprisingly, the HML approach identified a unique silicone elastomer formulation and printing parameters (bath concentration, ink viscosity, layer height, flow rate, and retraction distance) that had not been found from the EGO method (Abdollahi et al., 2018). Additionally, one significant benefit of HML is the ability to optimize parameters given very small data sets.

ML was also utilized in another recent study in which a set of parameters were tested to optimize print quality and speed via detection, prediction and smoothing modules to optimize local geometry of fused filament fabrication without expert intervention (Gardner et al., 2019). Each module was connected via initial training and continued learning to optimize the print time and quality. In addition to optimizing local parameters, hardware responses were accounted for to improve the quality of the final product. This work outlined an E2E (end-to-end) tool for integrating ML into the 3D printing process to correct visual flaws, which was augmented to training data.

The approaches discussed in this section all strive to promote print fidelity, a necessary 3DBP characteristic that defines a construct's ability to accurately reflect its blueprint.

### Efforts to Enhance Biomimicry in 3DBP

The optimization of biomimicry aims to find the biomimetic combination of print parameter values that best promote the construct's biological, mechanical, and rheological likeness to the native tissue. Currently, few bioinks have been capable of matching these necessary properties required for production of targeted tissue by 3DBP (Gungor-Ozkerim et al., 2018). However, there have been gradual and persistent efforts in improving bioinks and gaining critical information to promote biomimicry: (i) formulating a new bioink including therapeutic proteins to direct rapid migration of endothelial cells in a 3DBP object (Peak et al., 2019), (ii) emphasizing the need for coordinated spatial, biological and synthetic environmental cues to direct development of cells for tissue growth (Takebe and Wells, 2019), (iii) defining the transcriptional responses associated with stiffness, stress relaxation, and ligand density for stem cell differentiation and proliferation (Darnell et al., 2018), (iv) aiming to find the ideal biomimetic bioink by evaluating 3DBP structures made from different bioinks based on cell sedimentation, cell viability during extrusion, and cell viability after ink curing (Dubbin et al., 2017), and (v) combining multi-material bioinks to determine the optimal blend that can improve cell viability and spatial distribution via the crosslinking abilities of three different biopolymers (alginate, gelatin, and Matrigel) (Berg et al., 2018).

Another attempt to promote biomimicry showed that porosity and flow conditions were modulated to simulate shear gradients within solid tumors (Trachtenberg et al., 2018). The authors investigated the combination of scaffolds composed of pore size gradient with flow perfusion bioreactors to achieve complex, intrascaffold shear stress environments that can elicit a gradient phenotypic response in tumor cells. Scaffold porosity affected cell growth under flow conditions in that cells in the top layer experienced the highest level of shear stress, and the pressure drop within lower layers likely caused a decline in shear stress. In a similar case, optimization of pore size, porosity, and interconnectivity as a function of structural permeability and mechanics as well as material composition has been exemplified in bone tissue engineering (Diaz-Gomez et al., 2019). Layer-independent structures were created with high interconnectivity, thus without introducing weak points in the welding between segments. With printing resolution around 100–150 μm range, polycaprolactone-hyaluronic acid composite structures reached a compressive modulus around 126.2 ± 7.6 MPa attainable from human trabecular bone (50–150 MPa).

To produce bone scaffolds with desirable rates of degradation, finite element analysis (FEA) with GA was utilized to identify the diameter and spacing of PLGA [poly(lactic-co-glycolic acid)] scaffold that best promotes the required scaffold degradation profile (Heljak et al., 2017). The initial set of scaffold structures was generated randomly, and then each generated structure was evaluated using FEA. Based on the values of the fitness function, the probability of an individual being selected for the next GA iteration was calculated. The more fit an individual, the higher the probability of selection. Those individuals selected were subjected to genetic operators such as cross-over and mutation with a given probability. The cross-over and mutation operations resulted in a new set of scaffold structures to be evaluated in the following iteration. The GA was stopped after the max number of iterations was achieved. The overarching goal of the project was to design scaffolds that display the required stiffness at each stage of degradation. Thus, by combining FEA and GA, the authors were able to select the scaffold diameter and spacing that would achieve desired kinetics of polymer degradation.

The research discussed in this section highlights efforts toward promoting biomimicry, a critical consideration in 3DBP that defines a construct's ability to reflect the biological, mechanical, and rheological characteristics of its target tissue.

### Integrated Approaches to Optimization of Print Fidelity and Biomimicry in 3DBP

Integrated approaches that incorporate both print fidelity and biomimicry are necessary because if a construct has high print fidelity but is not able to serve its biological function, then it is useless in a clinical TE setting. Similarly, if a construct functions well from a biological standpoint but does not match the intended blueprint, then the construct might not physically fit into the space where it is needed and hence, would also not be useful in a clinical setting.

One study investigated a simple optimization method for 3DBP that considered one element of print fidelity and one element of biomimicry with a parameter optimization index (POI), which relied on the minimization of line thickness and shear stress to optimize the final construct (Webb and Doyle, 2017). Considering the following print parameters—print speed, nozzle diameter, and pressure—each of the 72 combinations of print parameters was assessed on line thickness and theoretical shear stress and given a POI value. The POI values were presumed to correspond with the ability of each combination to maximize print fidelity without sacrificing cell viability. A later study expanded the use of a POI to optimize fidelity and biomimicry via strand thickness, percent print accuracy, compressive modulus, and percent cell viability (Giuseppe et al., 2018).

To enhance functionality of bone tissue scaffolds, one research group attempted to optimize the scaffold topology and material properties with two different engineering strategies (Uth et al., 2017). The authors found the optimal component of nano-hydroxyapatite (nHA) content (30%) in the PLGA/collagen type I scaffold and the optimal strand diameter of 460 μm with both I-optimal, split-plot DOE (to minimize the average prediction variance over the design space) and COMSOL-based FEA. However, the two different optimization strategies showed disagreement in strand spacing of 908 μm with DOE and of 601 μm with FEA. The two optimization strategies resulted in similar scaffold porosity, but the prediction of compressive modulus was substantially poor (51% DOE and 21% FEA) partly due to the lack of accounting microtopology on the surface of scaffolds in the optimization strategies. The same I-optimal design was employed to fabricate 3D bio-printed bone scaffolds made of PLGA/nHA composites with compressive moduli exceeding 5 MPa and >85% porosity (Yousefi et al., 2019). This approach resulted in generating scaffolds with a porosity of 89% and a compressive modulus of 5.1 MPa with 10% (w/v) PLGA and 10% (w/v) nHA.

The studies discussed in this section begin to consider both print fidelity and biomimicry regarding parameter optimization; this research lays a foundation to continue to strive toward MI-based systematic approaches for creating patient-specific 3DBP tissue constructs.

## Conclusions and Outlook

As MI-based approaches emerge, a few cases utilized ML approaches to accelerate the rate of production in 3D printing with fewer errors, artifacts and bias. The majority of printed features are based on simplified or artificial patterns that are less relevant to tissue or organ architectures. A blueprint was created from tissues to fabricate engineered cardiac muscle (Gao et al., 2017) and the specific geometry of the heart was obtained with medical imaging modalities to construct life-size organs via 3DBP (Lee et al., 2019).

While the limitations of ML are easily found in many potential applications of 3DBP and TE, the field of ML has a rich history for solutions to tackle such limitations. For example, uncertainty analysis has been an active area with Bayesian learning. To improve the interpretability of a learned model, a number of cases for the model and variable selection are found in literature (Hastie et al., 2013). Despite significant challenges associated with such fundamental issues, we strongly believe that recent advances particularly in the domain of DL (such as incorporating sophisticated statistical models and other orthogonal approaches, namely Reinforcement Learning) could shed light on finding novel practical solutions. Again, it is the authors' anticipation that ultimately the systematic framework for a TE blueprint needs to include such capabilities.

It is our intention to emphasize the term of MI to be used for the future direction to overcome many existing problems with “the conventional ML,” including the uncertainty quantification, induction bias, and machine reasoning. Elaborating this perspective with an unbiased manner is beyond the scope of the manuscript prepared for a mini review. Nevertheless, the domain of ML is evolving fast, primarily motivated to deal with the new challenges of Big Data problems and high-throughput experiments. Thanks to recent advances in DL and other associated ML methods, many limitations, previously considered as roadblocks, are effectively or practically resolved.

MI processes are no longer regarded as a black-box model, but rather processes that can be explained with expected behaviors (Gilpin et al., 2018). Thus, each process depicted in Figure 1 can be understood better as MI matures over time. The more challenging task is to integrate both approaches—generating a regenerative blueprint and optimizing printing parameters—in an E2E 3DBP process without (significant) human intervention. Such progress potentiates the development of customized and life-size tissue or organ replacement in the near future via MI-guided 3DBP.

## Author Contributions

JJ and JK conceived the overall topics of discussion. All authors wrote, read, and approved the final manuscript.

### Conflict of Interest

The authors declare that the research was conducted in the absence of any commercial or financial relationships that could be construed as a potential conflict of interest.
