# Supplementary material for: Engineering Tissue Fabrication With Machine Intelligence: Generating a Blueprint for Regeneration
Source: Front Bioeng Biotechnol. 2020 Jan 10;7:443. doi: 10.3389/fbioe.2019.00443 (PMC6967031; doi:10.3389/fbioe.2019.00443)
Supplement: Supplementary file 1 [file Table_1.DOCX]

**Supplementary Information**

**Engineering tissue fabrication with machine intelligence: generating a blueprint for regeneration**

Joohyun Kim^1^, Jane A. McKee^2^, Jake J. Fontenot^2^, and Jangwook P. Jung^2^

1. Center for Computation Technology, Louisiana State University, Baton Rouge, LA, USA

2. Department of Biological Engineering, Louisiana State University, Baton Rouge, LA, USA

**Table S1.** Optimization approaches of increasing print fidelity

| **Type of Printing** | **Optimization Features** | **Parameters** | **Response variables** | **Reference** |
| --- | --- | --- | --- | --- |
| Low temperature, deposition manufacturing, 3D bioprinting |  | Extrusion rate, polymer solution concentration | Resolution, printed line continuity, wall thickness, visual similarity to blueprint | (Wang et al., 2017) |
| Direct melt extrusion bioprinting |  | Polymer composition (ratio of lactic acid to glycolic acid, molecular weight of the composition, organic end cap), fiber diameter, temperature, pressure, print pattern, print speed | Visual comparison to the CAD model | (Guo et al., 2017) |
| Extrusion-based bioprinting | Expert-guided optimization (EGO) method | Bath type, bath concentration, ink type, bath pH, ink concentration, bath stirring time, ink curing time, printing speed, filament packing density, layer height, tower, comb, retraction distance, solid surface thickness, infill pattern, grid extra overlap, infill solidity, | Stringiness (for printed cylinder), infill (for printed cylinder), layer fusion (for printed cylinder), bottom quality (for printed cube), wall fusion (for printed cube) | (Abdollahi et al., 2018) |
| Extrusion-based bioprinting | Hierarchical machine learning (HML) | Bath material, bath concentration, ink flow speed, needle diameter, ink material, retraction distance | Stringiness, infill, later fusion | (Menon et al., 2019) |
| Piezoelectric drop-on-demand (DOD) bioprinting | Multi-objective optimization (MOO) method | Applied voltage, viscosity of bioink, surface tension of bioink, nozzle radius | Printing precision and stability, satellite formation, droplet deformation | (Shi et al., 2019) |
| Fused filament fabrication 3D printing | Machine learning technique | Nozzle temperature, bed temperature, print speed, extrusion multiplier, fan speed, hardware responses | Print quality and print speed | (Gardner et al., 2019) |
| Rapid robotic free-form printing | Machine learning algorithm (genetic algorithm and gradient based algorithm) | Link one angular velocity, link two angular velocity, link three angular velocity, dispensing velocity, link one, link two, link three, surrounding cooling fluid velocity, surrounding convective cooling coefficient, volume fraction of phases 2, phase 1 mass density, phase 2 mass density, temperature of the robot, phase 1 heat capacity of the droplet, phase 2 heat capacity of the droplet, ambient temperature, droplet radius, phase 1 thermal conductivity, phase 2 thermal conductivity, pixel charge, pixel grid, per unit volume charge for phase 1, per unit volume charge for phase 2, radiative efficiency | The difference between the desired and generated patterns | (Zohdi, 2019) |

**Table S2.** Optimization approaches of enhancing biomimicry

| **Type of Printing** | **Optimization Features** | **Parameters** | **Response variables** | **Reference** |
| --- | --- | --- | --- | --- |
| Extrusion-based bioprinting |  | Bioink type | Cell sedimentation, cell viability during extrusion, cell viability after ink curing | (Dubbin et al., 2017) |
| Extrusion-based bioprinting |  | Combinations of biopolymer concentrations | Cell viability, cell spatial distribution | (Berg et al., 2018) |
| Extrusion-based bioprinting |  | Scaffold porosity | Cell growth | (Trachtenberg et al., 2018) |
| 3D bioplotting |  | Pore size, porosity, interconnectivity, material composition | Structural permeability and mechanics | (Diaz-Gomez et al., 2019) |
| Fused deposition modeling (FDM) | Finite element analysis (FEA) with genetic algorithm (GA) | Diameter and spacing of PLGA scaffold | Scaffold degradation | (Heljak et al., 2017) |

**Table S3.** Integrated approaches of optimizing print fidelity and biomimicry

| **Type of Printing** | **Optimization Features** | **Print accuracy optimization** | | **Functional performance optimization** | | **Reference** |
| --- | --- | --- | --- | --- | --- | --- |
|  |  | **Parameters** | **Response variables** | **Parameters** | **Response variables** |  |
| Extrusion-based bioprinting |  | Travel feed rate, nozzle diameter, GelMA concentration, extrusion pressure | Line thickness, line quality (continuity of flow and consistency of thickness) | GelMA concentrations, photoinitiator (LAP) concentrations | Young’s modulus | (Ersumo et al., 2016) |
| Extrusion-based bioprinting |  | Printing pressure, feed rate, printing height | Line thickness | Line spacing, printing direction with respect to tensile loading, addition of cells, bioink type | Line to line adhesion (ultimate stress, ultimate strain, secant modulus), rheological characteristics (shear behavior and shear recovery) | (Kesti et al., 2016) |
| Extrusion-based bioprinting | Parameter optimization index (POI) | Print speed, nozzle diameter, pressure | Line thickness | Print speed, nozzle diameter, pressure | Shear stress (assumed to have a direct negative impact on cell viability) | (Webb and Doyle, 2017) |
| Extrusion-based bioprinting | Parameter optimisation index (POI) | Bioink composition (alginate concentration and gelatin concentration), needle gauge, printing pressure | Strand thickness, percentage printing accuracy | Crosslinking time, storage condition, bioink composition (alginate concentration and gelatin concentration) | Compressive modulus, percent viability of encapsulated mesenchymal stem cells | (Giuseppe et al., 2018) |
| 3D inkjet printing |  | Voltage and pulse length | Geometric alignment and homogeneity of drops | Bioink composition | Cell attachment and growth | (Negro et al., 2018) |
| 3D Bioplotting | I-optimal, split-plot DOE, and COMSOL-based finite element analysis (FEA) | Scaffold composition, strand diameter, strand spacing | Scaffold topology | Scaffold composition, strand diameter, strand spacing | Material properties | (Uth et al., 2017) |
| Additive manufacturing and thermally induced phase separation (TIPS) | I-optimal DoE and the response surface analysis | PEG strand diameter, PLGA concentration, nHA content, TIPS temperature | Scaffold thickness | PEG strand diameter, PLGA concentration, nHA content, TIPS temperature | Porosity, compressive modulus | (Yousefi et al., 2019) |

**References**

Abdollahi, S., Davis, A., Miller, J.H., and Feinberg, A.W. (2018). Expert-guided optimization for 3D printing of soft and liquid materials. *PLoS One* 13**,** e0194890.

Berg, J., Hiller, T., Kissner, M.S., Qazi, T.H., Duda, G.N., Hocke, A.C., Hippenstiel, S., Elomaa, L., Weinhart, M., Fahrenson, C., and Kurreck, J. (2018). Optimization of cell-laden bioinks for 3D bioprinting and efficient infection with influenza A virus. *Scientific Reports* 8**,** 13877.

Diaz-Gomez, L., Kontoyiannis, P.D., Melchiorri, A.J., and Mikos, A.G. (2019). Three-dimensional printing of tissue engineering scaffolds with horizontal pore and composition gradients. *Tissue Engineering Part C, Methods* 25**,** 411-420.

Dubbin, K., Tabet, A., and Heilshorn, S.C. (2017). Quantitative criteria to benchmark new and existing bio-inks for cell compatibility. *Biofabrication* 9**,** 044102.

Ersumo, N., Witherel, C.E., and Spiller, K.L. (2016). Differences in time-dependent mechanical properties between extruded and molded hydrogels. *Biofabrication* 8**,** 035012.

Gardner, J.M., Hunt, K.A., Ebel, A.B., Rose, E.S., Zylich, S.C., Jensen, B.D., Wise, K.E., Siochi, E.J., and Sauti, G. (2019). Machines as craftsmen: Localized parameter setting optimization for fused filament fabrication 3D printing. *Advanced Materials Technologies* 4**,** 1800653.

Giuseppe, M.D., Law, N., Webb, B., A. Macrae, R., Liew, L.J., Sercombe, T.B., Dilley, R.J., and Doyle, B.J. (2018). Mechanical behaviour of alginate-gelatin hydrogels for 3D bioprinting. *Journal of the Mechanical Behavior of Biomedical Materials* 79**,** 150-157.

Guo, T., Holzberg, T.R., Lim, C.G., Gao, F., Gargava, A., Trachtenberg, J.E., Mikos, A.G., and Fisher, J.P. (2017). 3D printing PLGA: a quantitative examination of the effects of polymer composition and printing parameters on print resolution. *Biofabrication* 9**,** 024101.

Heljak, M.K., Kurzydlowski, K.J., and Swieszkowski, W. (2017). Computer aided design of architecture of degradable tissue engineering scaffolds. *Computer Methods in Biomechanics and Biomedical Engineering* 20**,** 1623-1632.

Kesti, M., Fisch, P., Pensalfini, M., Mazza, E., and Zenobi, M. (2016). Guidelines for standardization of bioprinting: A systematic study of process parameters and their effect on bioprinted structures. *BioNanoMaterials* 17**,** 193–204.

Menon, A., Póczos, B., Feinberg, A.W., and Washburn, N.R. (2019). Optimization of silicone 3D printing with hierarchical machine learning. *3D Printing and Additive Manufacturing* 6**,** 181-189.

Negro, A., Cherbuin, T., and Lutolf, M.P. (2018). 3D inkjet printing of complex, cell-laden hydrogel structures. *Scientific Reports* 8**,** 17099.

Shi, J., Song, J., Song, B., and Lu, W.F. (2019). Multi-objective optimization design through machine learning for drop-on-demand bioprinting. *Engineering* 5**,** 586-593.

Trachtenberg, J.E., Santoro, M., Williams, C., Piard, C.M., Smith, B.T., Placone, J.K., Menegaz, B.A., Molina, E.R., Lamhamedi-Cherradi, S.-E., Ludwig, J.A., Sikavitsas, V.I., Fisher, J.P., and Mikos, A.G. (2018). Effects of shear stress gradients on Ewing sarcoma cells using 3D printed scaffolds and flow perfusion. *ACS Biomaterials Science & Engineering* 4**,** 347-356.

Uth, N., Mueller, J., Smucker, B., and Yousefi, A.M. (2017). Validation of scaffold design optimization in bone tissue engineering: finite element modeling versus designed experiments. *Biofabrication* 9**,** 015023.

Wang, X., Rijff, B.L., and Khang, G. (2017). A building-block approach to 3D printing a multichannel, organ-regenerative scaffold. *Jouranl of Tissue Engineering and Regenerative Medicine* 11**,** 1403-1411.

Webb, B., and Doyle, B.J. (2017). Parameter optimization for 3D bioprinting of hydrogels. *Bioprinting* 8**,** 8-12.

Yousefi, A.-M., Liu, J., Sheppard, R., Koo, S., Silverstein, J., Zhang, J., and James, P.F. (2019). I-optimal design of hierarchical 3D scaffolds produced by combining additive manufacturing and thermally induced phase separation. *ACS Applied Bio Materials* 2**,** 685-696.

Zohdi, T.I. (2019). Electrodynamic machine-learning-enhanced fault-tolerance of robotic free-form printing of complex mixtures. *Computational Mechanics* 63**,** 913-929.
